# Psychoeducation for adult ADHD: a scoping review about characteristics, patient involvement, and content

**DOI:** 10.1186/s12888-024-05530-8

**Published:** 2024-01-25

**Authors:** Henrik Pedersen, Tatiana Skliarova, Sindre Andre Pedersen, Rolf W. Gråwe, Audun Havnen, Mariela L. Lara-Cabrera

**Affiliations:** 1 Department of Mental Health, Faculty of Medicine and Health Sciences, Norwegian University of Science and Technology (NTNU), Trondheim, Norway; 2 Division of Psychiatry, Nidaros Community Mental Health Centre, St. Olavs University Hospital, Trondheim, Norway; 3 Library Section for Research Support, Data and Analysis, NTNU University Library, Norwegian University of Science and Technology (NTNU), Trondheim, Norway; 4 Department of Psychology, Norwegian University of Science and Technology (NTNU), Trondheim, Norway; 5 Division of Psychiatry, Nidelv Community Mental Health Centre, St. Olavs University Hospital, Trondheim, Norway

**Keywords:** Adult ADHD, ADHD, Scoping review, Patient education, Psychoeducation, attention-deficit/hyperactivity disorder, Hyperkinetic disorder, User involvement, Patient involvement

## Abstract

**Background:**

Psychosocial interventions such as psychoeducation are increasingly being used to treat adult ADHD, both as an alternative and as a supplement to pharmacotherapy. A thorough overview of the literature on psychoeducation for adult ADHD is lacking. The objectives of this scoping review were therefore to identify the characteristics of psychoeducation interventions designed for adults with ADHD, examine how the patient experience or perspective is considered during the intervention’s development and implementation, determine the typical themes covered, and explore how ‘psychoeducation’ is defined in these interventions.

**Methods:**

A comprehensive search was performed to identify records in MEDLINE, Embase, PsycINFO, Web of Science, Cochrane CENTRAL, AMED, and ClinicalTrials.gov. Two or more reviewers were included in every step of the screening process and the final selection of included studies. The Preferred Reporting Items for Systematic reviews and Meta-Analyses extension for Scoping Reviews (PRISMA-ScR) checklist (Supplementary Material 1) was used to report the results, and the framework developed by Arksey and O’Malley was used as a guide throughout the scoping process.

**Results:**

A total of 2121 records were identified through the literature search. After screening and full-text analysis, ten studies were included for final analysis. Most studies were conducted in Europe and followed a group format. Seven main themes were identified: Information about the diagnosis, treatment options, somatic health and ADHD, the insider perspective, ADHD and social life, coping and psychological skills, and ADHD and work. There was significant overlap in themes covered, but coverage of each theme varied. Themes deemed important by newer research, such as sexuality and gender-specific issues, were missing. Only one intervention involved patients in its development and implementation, and two interventions involved family members. There was variation in how psychoeducation was defined in the included studies, and the implications of this are discussed.

**Conclusion:**

The literature on psychoeducation for adult ADHD is not ready for any systematic effect estimation. Before such estimations are conducted, a shared understanding and definition of psychoeducation are needed. The involvement of end users in the development and delivery of interventions may aid reach this goal but results from this review indicate that such practices are rare.

**Supplementary Information:**

The online version contains supplementary material available at 10.1186/s12888-024-05530-8.

## Background

Attention deficit hyperactivity disorder (ADHD) is a neurodevelopmental disorder characterized by persistent problems of inattention, hyperactivity, and impulsivity, with a debut in childhood [1]. While some who met diagnostic criteria as children or adolescents no longer meet diagnostic criteria in adulthood [2], it is estimated that about 2.5% of adults have ADHD worldwide [3, 4]. People diagnosed with ADHD often display secondary psychiatric problems, with 80% having a concurrent psychiatric diagnosis [5, 6]. Additionally, the diagnosis is associated with a range of other negative outcomes, including lower academic and occupational performance, higher risk of somatic disease, accidents, criminal behaviour, and suicide [7, 8]. Pharmacological interventions using stimulants have shown a good effect, making them the first line of treatment [9]. Nevertheless, it is estimated that as many as half of patients discontinue their medication [10], with the most common explanations being: no response to treatment, adverse effects, social stigma, patient attitude, and dosing inconvenience [11]. Furthermore, a recent study estimated that up to 58% of ADHD-diagnosed adults do not renew their prescription promptly enough to be considered consistently medicated [7]. Many clinicians and patients, therefore, opt for a non-pharmacological approach, as a substitute or parallel treatment to medication.

Non-pharmacological treatments have also shown promise in reducing symptoms and are considered the second line of ADHD treatment [9, 12, 13]. Systematic reviews of non-pharmacological or psychological interventions have practiced stringent inclusion criteria to assess effectiveness [12, 13]. The conclusion of these reviews, however, was that although non-pharmacological interventions show promise, the diversity of intervention types and heterogeneity of methods prohibited a proper effect estimation. This, in turn, calls for comprehensive reviews of more specific non-pharmacological interventions to get a complete picture of the currently available literature.

Psychoeducation represents a promising group of non-pharmacological interventions, which can be defined as helping the patient cope with their disorder-related problems, by providing them and/or their caregivers with systematic and structured didactic information about the disorder and its treatments [14]. Providing accurate disease information to patients has become a central part of treatment in both somatic and psychiatric healthcare, increasing compliance adherence and treatment motivation [14]. In mental health research, systematic reviews have shown psychoeducation to be beneficial for people struggling with long-term illnesses such as schizophrenia [15] and bipolar disorder [16], when it is provided for the patients and/or their families.

In theory, there are no restrictions to how psychoeducation can be delivered, which do not limit psychoeducation to a one-on-one interaction between clinician and patient. Indeed, group-based interventions and psychoeducation through digital media or programs may aid the dissemination of relevant knowledge. However, much disorder-related information is available on the internet, with no systematic quality assurance. Research evaluating popular ADHD-specific videos on the websites YouTube, and TikTok, has concluded that most videos are misleading, and presented by lay individuals [17, 18]. Hence, a thorough description of existing psychoeducational interventions is necessary.

Only two reviews specifically aimed at investigating psychoeducation for adult ADHD have been conducted, to date. A rapid review published in 2016 included only three studies for full review [19]. No critical appraisal of these studies or further assessment was done, due to the low number of studies identified. A scoping review conducted in 2018, aimed to identify how the concept of psychoeducation was characterized by researchers in the context of ADHD treatment [20], found six papers published in English covering psychoeducation for adults with ADHD. Out of these six, only two were intervention studies.

Considering all this, we argue that an updated, and more thorough literature review, is warranted for multiple reasons. First, the studies of psychoeducation interventions included in the previous reviews, only partly overlap [12, 13, 19, 20], indicating that the inclusion criteria were too stringent to include all psychoeducation articles, not getting a complete overview of the literature. Hence, a scoping review covering a broader range of psychoeducation interventions adopted in adults with ADHD is warranted. Second, a thorough description of existing interventions is necessary to determine what aspects of psychoeducation interventions are effective and relevant. Currently, no such overview exists. Third, attending to the patient experience and involving end users in the development and delivery of non-pharmacological interventions have been acknowledged as important to ensure both relevancy and effectiveness [13, 14]. Lastly, several relevant primary studies may have been published since the last review in 2018.

Due to the inconclusive findings of previous reviews, and the objectives of the current study we chose to conduct a scoping review. The objectives of this scoping review were to (1) identify the characteristics of psychoeducation interventions designed for adults, (2) examine how the patient experience or perspective is taken into account during the development and implementation of these interventions, (3) determine the typical themes covered in psychoeducation provided to adults diagnosed with ADHD, and (4) explore how psychoeducation is defined in psychoeducation interventions for adults with ADHD.

## Methods

A scoping review aims to ‘map the key concepts contained in a research domain—their breadth, limits, and features—and the primary sources and types of available evidence [with the intent] to produce a quick, narrative, descriptive account of the scope of current literature addressing a key research question’ (p. 298) [21]. This review followed the guidelines outlined by Arksey and O’Malley and its later iteration by Levac et al. [22, 23], dividing the review process into six stages: identifying a research question, developing a search strategy, study selection, data charting, synthesis of findings, and consultation. Our results are reported according to the Preferred Reporting Items for Systematic reviews and Meta-Analyses extension for Scoping Reviews (PRISMA-ScR; Supplementary Material 1) [24].

### Identifying research questions

In this review, we have adopted the broad definition of psychoeducation presented by Ekhtiari et al. [14]. Psychoeducation is an intervention with systematic, structured, and didactic knowledge transfer about an illness and its treatment, integrating emotional and motivational aspects to enable patients to cope with the illness and to improve its treatment adherence and efficacy. Interventions focusing on ADHD were classified as psychoeducation in cases where more than half of the program included systematic, structured, and didactic transfer of knowledge concerning the condition. Furthermore, in this review, we defined ‘psychoeducation intervention for adults with ADHD’ as any psychoeducation intervention where the goal is to reduce ADHD or concurrent secondary psychiatric symptoms and heighten everyday functioning, treatment adherence, or quality of life– directly or indirectly– in people over the age of 18 who have been diagnosed with ADHD. Our definition was broad, to ensure the consideration of a wide range of studies.

We developed the following specific research questions to address this review’s primary objective. What are the characteristics of psychoeducation interventions for adults with ADHD? What themes does psychoeducation provided to adults with ADHD typically cover? And finally, how is the patient experience or perspective considered in the development and implementation of these interventions? Because the definition of psychoeducation is associated with the inclusion criteria, the types of interventions, and outcomes, a secondary objective in this review, which emerged during the review process, was to describe the diversity of definitions and topics addressed in the included studies.

### Identifying relevant studies

To identify relevant studies, a structured literature search was run in the bibliographic databases MEDLINE, Embase, PsycINFO, Web of Science, Cochrane library, AMED, and the register ClinicalTrials.gov. The search strategy involved two main concepts: ‘psychoeducation’ and ‘ADHD’. Relevant free-text terms associated with each concept were used consistently across the databases. Available synonyms were also incorporated in the concept in the various databases. Search terms associated with each main concept were first combined using the Boolean operator OR, before combining the two concepts using the operator AND. The literature search was last updated on June 6., 2022. A detailed description of the search strategy adopted in the various databases is available in Supplementary Material 2.

### Study selection

**Fig. 1:**
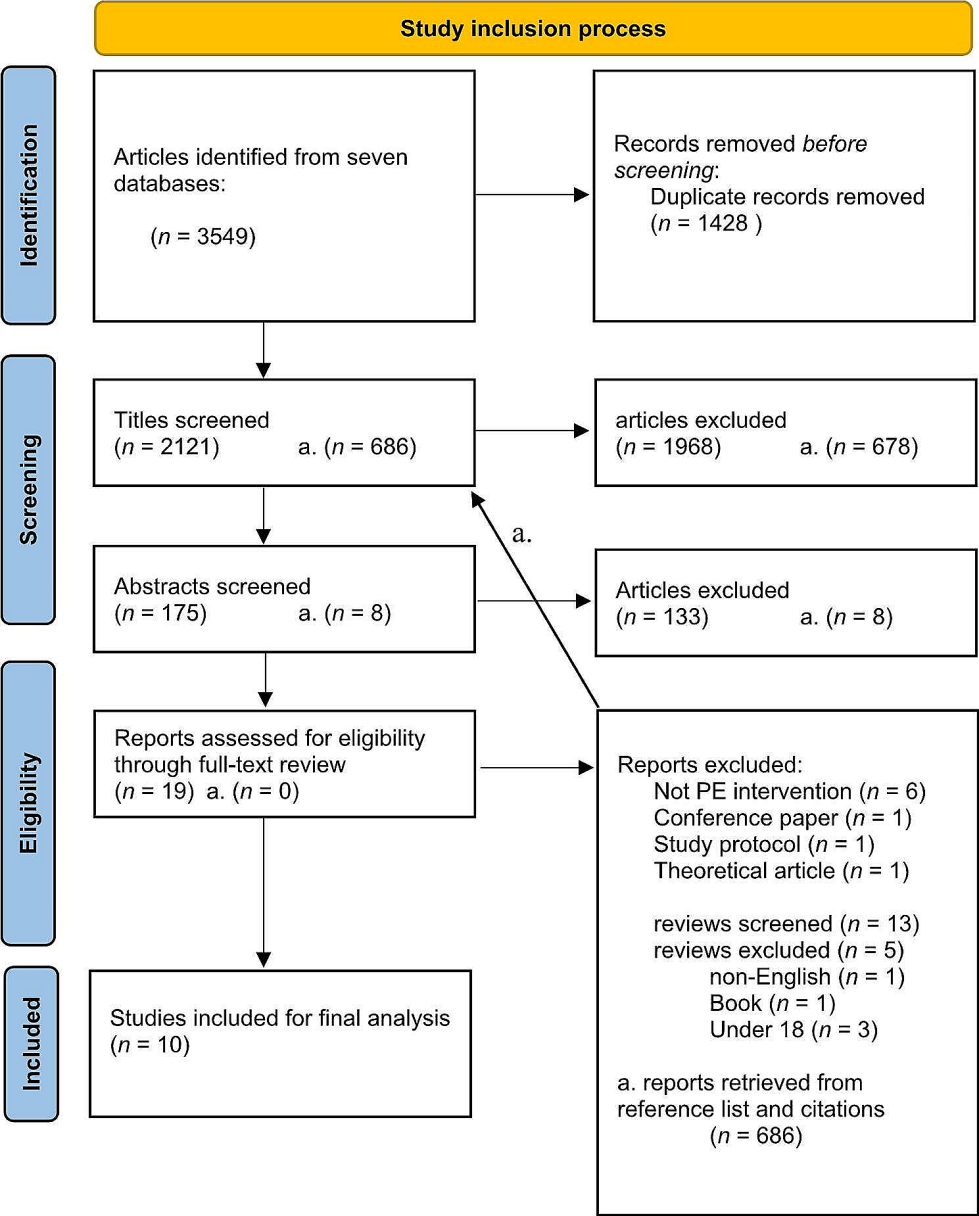
PRISMA flow diagram

The study selection process is outlined in Fig. 1. All records obtained from the databases were imported to EndNote 20 reference database software. Prior to screening, duplicate recodes were identified and removed. The screening of abstracts and titles and, subsequently, full-text evaluation to determine eligibility for inclusion was performed by a total of four independent reviewers. The first author, HP, screened all articles, while TS, AH, and MLL-C screened one-third each. This ensured that every article was evaluated twice. After each stage, the reviewers compared results and discussed potential discrepancies. In cases of disagreement after discussion, RWG was consulted as a fifth reviewer. For inclusion in the final analysis, a study had to be a peer-reviewed research paper evaluating a psychoeducation intervention for adults with ADHD. How this was ensured in practice is described below.

In the initial screening, only the titles were examined, and only articles written in English were included. Moreover, ‘ADHD’, ‘hyperkinetic disorder’ or other relevant terms had to be mentioned in the title. At this stage, books and book chapters; book reviews; editorials; opinion articles; comments on papers; conference papers, presentations, posters; and perspective articles were excluded. If the people diagnosed with ADHD were referred to as ‘children’, ‘adolescents’, ‘pupils’, ‘teenagers’, or ‘youths’ the study was excluded. Other age-restricted terms, such as ‘conduct disorder’, also lead to exclusion. Ambiguous terms such as ‘students’ or ‘young people’ were included for abstract screening.

A study was excluded if the title or abstract explicitly stated that the study participants were less than 18 years old. ‘Psychoeducation’, ‘patient education’, or related terms had to be mentioned in the title or abstract for inclusion. For example, a review titled ‘non-medical approaches for treating adult ADHD’ would have been included for further investigation despite psychoeducation not appearing in the title and possibly not in the abstract.

At this stage, the reference list, and citations of relevant review articles, reported in Google Scholar, were examined for additional studies, which was evaluated by the inclusion process already mentioned. Articles on guidelines and recommendations for the assessment and treatment of adults with ADHD were excluded. We cross-referenced registered clinical trials, acquired from the search, with published studies to ensure that relevant and completed research was not.

To be eligible for final analysis, a report had to be a research study, where at least one of the conditions evaluated was a psychoeducation intervention according to the definition above. At this stage, all four reviewers read the full-text articles and classified them as either included or excluded. An article was only included in the final analysis if a consensus was reached, defined as all four reviewers agreeing following discussion. If a consensus was not reached, RWG was consulted before a final decision was made.

### Data charting and synthesis

To see if the data charting form needed any refinements, two authors, HP and TS, independently read and extracted data from three of the studies included in our final analysis and then compared their work. Only minor language changes were made to the data charting tables, to ensure clarity, before data extraction ensued. Data from all the studies were then extracted separately, compared, and combined into a final table through active discussion. An overview of themes and definitions were obtained in the same manner, gathered independently from the studies themselves, or from the manuals used in the studies, compared, and combined through discussion. Data synthesis was based on the finalized tables and achieved through discussion among the authors and user representatives facilitated by the first author.

### Consultation: patient and public involvement

Two user representatives were involved throughout the scoping review. They collaborated in developing the idea of the review and were consulted regarding the scope of the review, when synthesizing the results, and when discussing aims for future research. Both representatives were so-called expert users, with over 15 years’ experience from working in non-profit user-driven support services. Both representatives have been regional board members in the Norwegian ADHD user organization. One was still an active board member when writing this article.

## Results

The search retrieved 3549 records, reduced to 2121 after removing duplicates. Initial screening of titles and abstracts identified 19 primary studies for full-text review and 13 reviews. Examination of the reference lists and citations of review articles led to an additional 686 records being screened, but no additional studies got past abstract screening. A consensus was reached through discussion on 15 of the 19 articles after full-text analysis, leading to four articles being evaluated by co-author RWG before further discussion and a final decision. Ultimately, ten articles were included in the final analysis. This process is presented in its entirety in Fig. 1. A list of the excluded articles after the full-text and their reason for exclusion is provided as Supplementary Material 3. Below are data extracted from the included articles summarized.

### Characteristics of published studies

A summary of the included studies is presented in Table 1. A majority of the studies were conducted in Europe [25–31] (*n* = 7). Two studies were conducted in Sweden [26, 27], two in Germany [25, 28], and one in Ireland [30], the Netherlands [29], and Spain [31] respectively. Outside of Europe, one study was conducted in Korea [32], one in the United States [33], and one in Brazil [34].

**Table 1:** Summary of interventions

Demographic information about participants						
Author, year	Name	Sample size	Country, setting	Age, mean (range)	Gender, women	Employment
Bachmann et al., 2018 [25]	mindfulness and psychoeducation on working memory in adult ADHD	Total *n* = 74 Final analysis *n* = 40 Mindfulness (MAP): *n* = 21 Psychoeducation (PE): *n* = 19	Germany, outpatient setting	MAP: 40.1 PE: 40.26	22 (55%)	Not reported
De Oliveira et al., 2018 [34]	Effectiveness of a psychoeducational booklet on ADHD in college students	Total *n* = 241 Health professionals: *n* = 25 ADHD in treatment: *n* = 35 General population: *n* = 181	Brazil, online survey	28.9	192 (79.7%)	Not reported
Hartung et al., 2022 [33]	Organizational and Study Skills Intervention for College Students with ADHD	Total *n* = 30	USA, university campus, two psychology training clinics	22.6 (18–32)	13 (43.3%)	Not reported
Hirvikoski et al., 2015 [26]	Psychoeducational groups for adults and their significant others (PEGASUS)	Total *n* = 108 *n* = 81 included in the final analysis (41 with ADHD, 40 significant others)	Sweden, two outpatient tertiary psychiatric clinic	Participants with ADHD 37.56, (20–63)	Participants with ADHD 15 (36.6%)	Participants with ADHD, full-time employed, studying, or parental leave: 24 (58.54%)
Hirvikoski et al., 2017 [27]	Psychoeducational groups for adults and their significant others (PEGASUS)	Total *n* = 179 ADHD: *n* = 87 significant others: *n* = 92 (SO) **Intervention**: ADHD: *n* = 48 SO: *n* = 49 **TAU**: ADHD: *n* = 39 SO: *n* = 43	Sweden, two outpatient tertiary psychiatric clinics	Participants with ADHD Intervention (INT): 38.6 (19–59) TAU: 38.2 (20–65)	Participants with ADHD 52 (59.8%)	Participants with ADHD, Full-time employed or studying INT 36 (75.0%) TAU 26 (66.7%)
Hoxhaj et al., 2018 [28]	Mindfulness group vs. psychoeducation group	Total *n* = 81 Mindfulness group (MAP): *n* = 41 Psychoeducation (PE): *n* = 40	Germany, outpatient clinic	MAP: 40.51 PE: 38.5	MAP: 23 (56%) PE (19 (47.5%)	Full-time employed, studying, or in an apprenticeship: MAP: 35 (85.4%) PE: 32 (80%)
In de Braek et al., 2017 [29]	Goal management training and psychoeducation vs. group psychoeducation	Total *n* = 27 Goal management training and psychoeducation (GMT+): *n* = 12 Psychoeducation (PE): *n* = 15	Netherlands, outpatient clinic	GMT+: 35.5 PE: 37.9	GMT+: 5 (41.7%) PE: 5 (33.3%)	Not reported
Jang et al., 2021 [32]	Chatbot (‘Todaki’) delivering psychoeducation and brief CBT	Total *n* = 46 Chatbot *n* = 23 Control *n* = 23	Korea, outpatient clinic	Chatbot: 26.7 Control 22.87	Chatbot 13 (57%) Control: 13(57%)	Full-time employed or studying: Chatbot: 10 (45%) Control: 7 (30%)
Salomone et al., 2012 [30]	A biofeedback-based psychoeducation program	Total *n* = 21 Self-alert Training (SAT) *n* = 10 Attention Training (AT) *n* = 11	Ireland, outpatient clinic	SAT: 32.4 AT: 33.2	Not reported	Not reported
Vidal et al., 2013 [31]	Group psychoeducation vs. group CBT	Total *n* = 32 Psychoeducation (PE): *n* = 17 Group CBT: *n* = 15	Spain, outpatient clinic	PE: 39.53 Group CBT: 39.4	PE: 11 (64.7%) Group CBT: 6 (40%)	Full-time employed or studying: PE:13 (76.47%) Group CBT: 11 (73.33%)
**Summary of study design**						
**Author, year**	**Name**	**Study design**	**Description of the intervention**	**Description of Comparison**	**Delivered by**	**Duration and structure**
Bachmann et al., (2018) [25]	Comparative study of mindfulness and psychoeducation	RCT, blinded assessor	Manualized group-based mindfulness (Zylowska et al., 2008) [59]	Based on highly structured psychoeducation manual (D’Amelio, 2009) [60]	Health profesionals	8 weekly group sessions of 2.5 h
De Oliveira et al., 2018 [34]	Effectiveness of a psychoeducational booklet on ADHD in college students	An online survey, of three populations	A brief online booklet with goals of increasing ADHD knowledge and removing barriers to seeking help	a. People in treatment for ADHD b. Professionals working with ADHD c. Population sample	online	Booklet length: 50 pages of illustrations and light text
Hartung et al., 2022 [33]	Organizational and Study Skills Intervention for College Students with ADHD	Open trial	Manualized individual and group sessions of skills training and psychoeducation	No comparison or control group	Graduate student therapists	6 group sessions and 2 individual sessions in 6 weeks. 3 optional sessions were offered to participants as well. Length not specified
Hirvikoski et al., 2015 [27]	Psychoeducational groups for adults with ADHD and their significant others (PEGASUS)	Open feasibility trial	Manualized Highly structured psychoeducation groups, in groups of 20–30 individuals	No comparison or control group	Both health professionals and individuals with ADHD	8 weekly group sessions of 2.5 h
Hirvikoski et al., 2017 [26]	Psychoeducational groups for adults with ADHD and their significant others (PEGASUS)	RCT, waitlist control	Manualized Highly structured psychoeducation groups, in groups of 20–30 individuals	Treatment as usual; This was not monitored in this study. TAU group received PEGASUS the following semester.	Both health professionals and individuals with ADHD	8 weekly group sessions of 2.5 h
Hoxhaj et al., 2018 [28]	Mindfulness group vs. psychoeducation group	comparative RCT, blind assessor	Manualized group-based mindfulness (Zylowska et al., 2008) [59]	Based on highly structured Psychoeducation manual (D’Amelio, 2009) [60]	Health professionals	8 weekly 2.5-hour group sessions
In de Braek et al., 2017 [29]	Goal management training and psychoeducation vs. group psychoeducation	Comparative RCT	Goal management training and psychoeducation (GMT+): Manualized training to improve executive functioning (Levine, 2000). In groups of 6 to 8 individuals.	Only psychoeducation; same material but without training.	Neuropsychologists	12 weekly two-hour group sessions, one individual, eleven group sessions
Jang et al., 2021 [32]	Chatbot (‘Todaki’). psychoeducation and brief CBT	Randomised controlled feasibility trial	Interactive chatbot ‘Todaki’	Bibliotherapy	Mobile app	4 weeks
Salomone et al., 2012 [30]	A biofeedback-based psychoeducation program	Randomised comparative trial	Self-alert training (SAT) Psychoeducation about attention, 20 min of biofeedback, and 10 min of computerized exercises daily.	Attention training (AT) psychoeducation about attention and 10 min of computerized sessions daily.	‘trainers’	2 sessions with a trainer, length not specified, in addition to a daily training regime over 5 weeks
Vidal et al., 2013 [31]	Group psychoeducation vs. group CBT	Randomised comparative pilot study	Structured didactic group psychoeducation.	Group CBT, based on Ramos-Quiroga et al. (2008)	Health profesionals	12 weekly two-hour group sessions over 3 months

Note. ADHD: Attention Deficit/Hyperactivity disorder; AT: Attention training; CBT: Cognitive behavioural therapy; RCT: randomised controlled trial; GMT+: goal management training and psychoeducation; MAP: Mindfulness training; PE: Psychoeducation; SAT: Self alert training; SO: Significant other; TAU: Treatment as usual

Seven studies were done at outpatient clinics [25–31], the remaining ones at student health-care services at a university [33]. Two studies were carried out online. One of these also recruited their participants online [34], while the other recruited from an outpatient clinic [32] before testing their chatbot. Most of the studies reported demographic information such as education level [25–28, 31–35] (*n* = 9) and employment status [26–28, 31, 32, 35] (*n* = 7), but few reported on marital status [28, 31, 35] (*n* = 3), and only one reported on ethnicity [33]. With regards to medication, most studies included and reported their participants’ active medication use. Three studies did not report medication use [30, 33, 34], and one study had psychostimulant use as an exclusion criterion [28]. Two studies did not provide any information about concurrent psychiatric issues in their sample [30, 34], the rest did assess for concurrent psychiatric disorders. Two studies had concurrent psychiatric issues as an exclusion criterion [29, 31]. De Oliveira et al. and Salomone et al. [30, 34] did not provide any information about the sex or gender of their participants. Aside from the two latter studies, 130 out of 256 participants with ADHD in the remaining studies were women (50.8%).

Seven of the studies were randomized controlled trials [25, 27–32]. Three studies used blind assessors at follow-up [25, 28, 29]. Six of the randomized trials had active controls, ranging from bibliotherapy [32] (*n* = 1) to weekly sessions that, in theory, would match the comparing condition in scope [25, 28–31] (*n* = 5). In terms of design regarding the last three studies, two interventions were open trials [26, 33], and one intervention had a quasi-experimental design [34].

Six studies evaluated a psychoeducation intervention as a primary goal [26, 27, 31–34]. One study compared two types of psychoeducation interventions [30]. Three studies used psychoeducation as an active control, two with the primary goal of evaluating a mindfulness intervention [25, 28], and the other, evaluating a goal management training intervention [29], a type of cognitive rehabilitation program.

With the exception of the quasi-experimental intervention evaluating a psychoeducation booklet [34], the length of the interventions varied from four [32] to 13 [25] weeks, the most common being eight weeks (*n* = 4). The most common format was group format (*n* = 7), with weekly sessions [25–29, 31, 33]. Most studies did not collect any follow-up data, and six studies did not do any measurement beyond post-intervention assessment [25, 30–34]. Two studies did follow-up measurements at three months [27, 29], and the remaining two did follow-up measurements at six months [26, 28].

### Patient involvement

Six face-to-face interventions [25, 28–31, 33] were delivered by health professionals or therapists in training. The remaining two, which both evaluated the PEGASUS intervention [26, 27], had a session where an individual with ADHD lectures about ‘living with ADHD’. This was also the only intervention where we were able to find information about patient involvement in the development of the intervention.

Five of the ten studies measured patients’ satisfaction as a way of attending to the experience and opinions of the participant about the intervention. This was the only way the patient experience was measured in the included studies. Of these five, two used an ad hoc measure created for the purpose of the study [33, 34]. The two studies that evaluated the PEGASUS intervention [26, 27] used a measure previously used to evaluate group cognitive behavioural therapy (CBT) for adults with ADHD [36]. Jang et al. [32] used a questionnaire that has previously been used to evaluate satisfaction with chatbots [37, 38].

### Content

Exploration of the content presented in the articles, and if provided, the manuals used, yielded seven main themes. Information about the diagnosis, treatment options, somatic health and ADHD, ADHD and social life, the insider perspective, practical and psychological skills to aid coping, and ADHD and work. The extent to which each theme was covered varied. For example, Hoxhaj et al. and Bachman et al. [25, 28], which both were based on the same modified version of a standardized manual [39], addressed in their first session ‘symptoms, causes, and treatments’ of ADHD. Other interventions divided these three topics into single sessions, or devoted whole sessions to different aspects of, for example, executive functioning (i.e., attention, memory, planning, or prioritizing). Some themes may also overlap, as description of lectures seem to suggest ways of coping with difficulties in these areas as well.

All interventions provided information about the diagnosis and its symptoms, the next most common theme was information about practical or psychological coping strategies. There was often more than one session devoted to some form of coping, varying from strategies to stay organized and structure one’s life, study skills, stress management, how to deal with failure and training of executive functions. Although this was a common theme, only one study explicitly mentioned actual in-session training [30]. Two studies explicitly mentioned that their intervention did not involve any form of in-session skills training [29, 31].

Most studies also covered information about different treatment options, both pharmacological and non-pharmacological. Only the PEGASUS intervention dedicated a session to somatic health, and one to the insider perspective [26, 27]. This was also the only intervention that informed about available support measures provided by the local social services that the participants may not know about. An overview of the interventions and to what degree they covered the main themes mentioned are presented in Table 2.

**Table 2:** Content of psychoeducation interventions for ADHD

Authors, year	Themes						
	ADHD diagnosis, symptoms, and functioning	Treatment options	Somatic health and ADHD	ADHD and Social life	Insider perspective	Practical and psychological skills	ADHD and work
Bachman et al., 2018 [25]; Hoxhaj et al., 2018 [28]	+	+		+		++	
De Oliveira et al., 2018 [34]	+	+					
Hartung, et al., 2022 [33]	+	+				++	
Hirvikoski et al. 2017; 2015 [26, 27]	+	+	+	+	+	+	+
In de Braek, 2017 [29]	++			+		++	+
Jang et al., 2021 [32]	+	+				++	
Salomone et al., 2012 [30]	+					++	
Vidal et al., 2013 [31]	++	+		+		++	

Note: A single plus indicates that the theme was covered in the intervention, and two pluses indicate a more comprehensive coverage relative to other topics; rows contain more than one study if the same manual was used

Indirectly related to content; The PEGASUS intervention demanded the participants bring a significant other to the sessions and the intervention was designed with that in mind [26, 27]. Vidal et al. had one session where participants could bring family, or a significant other if they pleased. All the other interventions were for patients only. Finally, information regarding the development of the different interventions was limited.

### The definition of psychoeducation

Four of the included studies provided a definition of ‘psychoeducation’ [25, 27, 31, 34], while an additional four studies provided an indirect definition by describing the goal of psychoeducation [26, 28, 29, 33]. Two studies did not provide any definition [30, 32]. Although all descriptions of psychoeducation involved some element of providing information, or alternatively, improving comprehension or giving insight, some included additional elements. Three emphasized the difference between psychoeducation and other types of interventions [25, 28, 31]. Two included learning skills in their definition [28, 29], in addition to providing information. Three studies [26–28], emphasized experience sharing, and mutual support among participants, implying that psychoeducation is a group experience. Two studies also included the involvement of significant others in their definition [26, 27]. All studies but one referred to the receivers of psychoeducation as patients (and potentially their significant others), this study defined psychoeducation as informing the public [34]. Full definitions and descriptions from all the studies are provided in Table 3.

**Table 3:** Definitions of ‘psychoeducation’ presented in the included papers

Authors, year	Definition of psychoeducation
Bachman et al., 2018 [25]	*[Psychoeducation] is an approach that aims at improving the patients’ understanding and awareness of the disorder; it can offer insight into past difficulties and can improve the patient’s general functioning (Vidal et al., 2013). [The] major objective [of psychoeducation] is to provide patients with information about their disorder. These characteristics distinguish [psychoeducation] from other psychological interventions that focus more on cognitive and behavioural changes, such as cognitive behavioural therapy methods involving cognitive restructuring, behavioural change, or mindfulness meditation practice.* (p.48)
De Oliveira et al., 2018 [34]	*Psychoeducation is the process of communicating relevant information to the* *population about a particular disorder (diagnosis, etiology, functioning), its treatment, and prognosis while seeking to clarify doubts and correct distorted beliefs*. (p.283)
Hartung, et al., 2022 [33]	No definition provided, but psychoeducation was indirectly described through its goal: *The psychoeducation module was included because emerging adults with ADHD often have a limited understanding of the disorder, particularly regarding evidence-based treatments that exist for it.* (p.414)
Hirvikoski et al. 2015 [26]	No definition was provided, but psychoeducation was indirectly described through its goal: *Psychoeducational interventions are aimed at empowering patients and their significant others with knowledge and directly ask patients to share in their own treatment (Hayes and Gantt 1992).* (p.90)
Hirvikoski et al. 2017 [27]	*Psychoeducation constitutes an approach to intervention providing information about ADHD and presents the opportunity to share experiences with people in a similar life situation, including the perspective of significant others. Importantly, and in contrast to most pharmacological and psychotherapeutical treatments, psychoeducation does not have the primary goal of reducing core symptoms, but aims at improving functional outcomes for the affected individual and to alleviate the burden of care on family members through collaborative management of everyday challenges (Dixon et al.,2001).* (p.142)
Hoxhaj et al., 2018 [28]	No definition provided, but psychoeducation was indirectly described through its goal: *The psychoeducational approach differs clearly from the [mindfulness training] concept with regard to topics and strategies*. (p.322) […] *The aim of the [psychoeducation] group (D’Amelio et al., 2009) is to provide information on the causes, symptoms and treatment options for ADHD in adulthood as well as the activation of organizational skills and stress management techniques, improving compliance, self-esteem and mutual support between the participants in everyday problems*. (p.323)
In de Braek, 2017 [29]	*One major addition to [goal management training] for ADHD patients concerns the nature of “psycho-education,” that is, an explanation of the various cognitive functions and the clinical picture of ADHD in adults in general.* (p.1132). *Because adults with ADHD often suffer from mood swings and low self-esteem, we added psychoeducation to [goal management training] to provide the patient with more insight into their condition. The aim of psychoeducation was to give the patients an additional tool to control their behavior and enable the selection of the most efficient coping strategy. The psychoeducation was concerned with various aspects of ADHD and various neurocognitive functions, like attention, memory, planning, distraction, and coping strategies, in particular.* (p.1131).
Jang et al., 2021 [32]	No definition was provided.
Salomone et al., 2012 [30]	No definition was provided.
Vidal et al., 2013 [31]	*Psychoeducation is another psychological approach different from CBT. This treatment is an intervention focused on the patients’ comprehension of their own disorder. Its objective is improving the patients’ understanding and awareness of the disease.* (p.894)

Note: Text in square brackets was added for clarity by the authors of this paper; References presented inside quotes in the table that are not referred to in the article are not included in this article’s reference list

## Discussion

This scoping review aimed to identify the characteristics of psychoeducation interventions designed for adults with ADHD, examine how the patient experience or perspective is considered during the interventions’ development and implementation, determine the typical themes covered, and explore how ‘psychoeducation’ is defined in these interventions.

Most studies were conducted in Europe, indicating that psychoeducation of this population may in large part be a European phenomenon. Most studies had a group format, which seems to be the case with non-pharmacological interventions for adult ADHD in general [13]. Only the PEGASUS intervention and Vidal et al. included significant others in their program [26, 27, 31]. This is inconsistent with the findings of an earlier scoping review, which included studies on children, adolescents, and adults [20] and, concluded that psychoeducation interventions most often were directed toward people important to the patient. Therefore, it seems as if the inclusion of family members or significant others is much more common when the person with ADHD is under 18.

### Patient involvement

PEGASUS was the only intervention developed and implemented with end users, and having former patients lecture in one of the sessions, indicating that this practice is rare in the context of treating adult ADHD. During recent decades, however, direct involvement of stakeholders outside of academia, such as end users, has become increasingly common in a range of research context [40], with some viewing such involvement as a prerequisite to ensure relevant and rigorous research, and to identify potential pitfalls when implementing successful interventions in local communities and routine clinical practice [40].

Indeed, during our consultation sessions user representatives pointed out that it seemed like most interventions were developed ‘in isolation’ without any connection to other local health, community, or social services offered. This isolation, in turn, has the potential to make the multimodal treatment recommended when treating adult ADHD [9] hard to achieve in practice. For example, a psychoeducation intervention may be effective, but afterward, some may also require debt counselling, academic support, or social evenings or lectures provided by user organizations. A proposed measure was to embed in interventions the opportunity for user organizations, expert users, or social workers to provide updated information about relevant local services outside of the health care system. As the results from this review reveal, ‘co-created’, or ‘co-delivered’ interventions are rare, and research on such interventions are minimal.

In terms of attending to the patient experience, half of the included studies measured patients’ satisfaction as a way of estimating acceptability and receiving patient feedback. Patient satisfaction is widely used in this context. However, all studies either used ad hoc satisfaction measures developed specifically for the study, or satisfaction measures only used a few previous studies. This is a concern raised by systematic reviews on patient satisfaction. Widely used, validated measures are a prerequisite for its measurement to be meaningful in itself [41], and when making inter-study comparisons [42, 43], as this requires a valid reference point.

Hirvikoski et al. [26, 27] measured the satisfaction of both significant others, and participants with ADHD were reported. However, Hirvikoski et al. [26] was the only study to provide information about satisfaction with individual sessions. Here, the participants were the most satisfied with the ‘living with ADHD’ lecture given by a former patient. No further analysis of satisfaction scores was done, besides reporting aggregated scores.

Reporting and analysing the satisfaction of different subgroups may be particularly important when evaluating interventions for disorders like ADHD, with a greater difference in symptom manifestation and type of struggles between the sexes [44]. In such cases, the intervention may be created around a stereotypical ADHD patient. Measuring patient satisfaction may help prevent this and provide valuable feedback when evaluating interventions on which elements may be the most useful.

### Content

There was relatively high overlap in the central themes covered, but the time devoted to each theme varied. All interventions gave patients information about the ADHD diagnosis and its symptoms, and most covered treatment options and information on practical and emotional coping strategies.

Contrary to our expectations, we were unable to find any studies on psychoeducation interventions for adults directly aimed towards increasing pharmacological treatment adherence, nor were any interventions directly aimed towards parents who are diagnosed with ADHD themselves. Therefore, if such information is routinely given in primary or specialized care, our results indicate that systematic evaluations of these interventions are lacking in the research literature.

Also noteworthy was the lack of mentions of gender-specific issues or other issues related to sexuality, or difficulties in sexual function related to the disorder. For example, ADHD has been found to be associated with a substantial higher risk of unplanned pregnancies, and risky sexual behaviours [45, 46]. Moreover, a recent systematic review about sexual health in ADHD [47], which argues that sexuality and sexual function in ADHD is an underexplored topic, found that sexual health among people with ADHD seems poor, with a tendency to both feel heightened sexual desire and worse performance than the general population. Our findings mirror the concerns highlighted in this review. Sex and sexuality, a central part of most intimate partner relationships, does not seem to be addressed when informing adults with ADHD about their diagnosis.

Although ADHD has traditionally been recognized as a predominantly male disorder, ADHD in girls and women is becoming more recognized [44], with some experts calling for gender-specific interventions for children and adolescents [48, 49], as current research suggests that the same symptoms lead to different struggles and outcomes at group level.

Little information was reported regarding the development of the different interventions. This makes it hard to know what type of stakeholders were involved during development, and on what basis the decisions regarding content coverage were made. None of the included studies described any theoretical frameworks in detail, or the foundation on which the intervention was based. One reason for this, may be that the ‘information’ presented in psychoeducation interventions may be viewed as atheoretical, in other words, as objective knowledge representing the current scientific consensus.

### The definition of psychoeducation

All definitions and descriptions provided by the included studies involved some kind of information transfer, although their emphases differed. Interestingly, if one strictly adhered to one of the definitions provided, this would exclude most, if not all, of the other included studies as psychoeducation interventions. There are also some inconsistencies within the articles. For example Hoxhaj et al. state that psychoeducation and mindfulness clearly differ [28], but subsequently remove certain exercises from the psychoeducation manual they used to define psychoeducation (p.322). This highlights the absence of a generally agreed-upon definition of ‘psychoeducation’ in the adult ADHD literature which is indicative of several problems.

First, when judging whether an intervention is a psychoeducation intervention, a demarcation problem emerges. Most interventions for adults with ADHD include some psychoeducation as part of the intervention [50] as it is regarded as a key component in, inter alia, ADHD counselling and ADHD-specific CBT [51]. However, some studies also report delivering psychoeducation in the style of a therapeutic paradigm. Examples of this include psychoeducation delivered in a motivational interview style [52, 53], and psychoeducational content available through short, simple digital CBT sessions provided by a chatbot [32].

Another demarcation problem arises concerning structure. For example, in this review a randomized controlled trial, evaluating internet-based support and coaching with complementary clinic visits [35], was excluded at the full-text review stage. This study reported that the participants were provided with individualized psychoeducation, but when compared to the definition above, we deemed the intervention to be individual coping-oriented counselling due to its unstructured nature.

Furthermore, the term psychoeducation is also sometimes used without further explanation. For example, two excluded studies from our initial search referred to TAU as ‘usually consisting of pharmacotherapy and/or psychoeducation’ [54, 55], but without monitoring the TAU group. It is therefore unclear what kind of psychoeducation is meant. Were this group provided with brief information from their general practitioner, a booklet, web addresses for user organizations’ web pages, or offered a 12-session psychoeducation program?

These issues may also reflect a problem with the definition of psychoeducation across diagnoses, how it is used more generally, and therefore going beyond the context where it has been examined here. However, no such conclusion can be made at this time based on the scope of this review and our results alone. Therefore, further investigations of the concept of psychoeducation in and across different diagnostic contexts are warranted.

Because the literature on psychoeducation on adults with ADHD is sparse, it is also safe to assume there are regional and national differences in ‘treatment as usual’, making comparisons even more difficult. For example, a study in Norway [56], (the resident nation of the authors), found that only about 20% of adults with ADHD reported being offered other treatment than medication. A larger, newer study [57], examining the living conditions of adults with ADHD in Norway, corroborates these results. Only 22% of participants that received treatment for ADHD in the last 12 months (*n* = 2923) recalled having been offered psychoeducation or cognitive therapy. Taken together, this makes it difficult to determine which interventions qualify as psychoeducation interventions, consequently this makes it challenging to compare different ‘psychoeducation’ interventions or systematically estimate its effectiveness when treating adult ADHD.

### Strengths and limitations

Our research employed a comprehensive and inclusive search strategy, coupled with broad inclusion criteria, which enabled us to encompass a wide range of studies relevant to our topic. The definition we adopted for ‘psychoeducation intervention’ was carefully considered, facilitating a focused and meaningful analysis within our research scope. However, despite our extensive approach, we acknowledge the possibility of having overlooked potentially relevant studies. This oversight could have affected our results, a limitation we must consider when interpreting our findings. Our reliance on our specific definition of ‘psychoeducation intervention’ also means that our review may not encompass broader interpretations of the concept, from which could influence the generalizability of our results. Furthermore, our review specifically focuses on psychoeducation for adults diagnosed with ADHD. The broader definition of psychoeducation across different mental health-, and diagnostic settings may warrant a separate, comprehensive review.

The exclusion of non-English language studies may have limited the diversity and representativeness of our dataset, potentially omitting valuable insights from non-English speaking regions. Additionally, our decision to not include grey literature (such as conference abstracts, theses, and non-peer-reviewed reports) in our search could have resulted in missing emerging research and innovative approaches not yet available in peer-reviewed journals. In light of these considerations, we suggest future studies might adopt a more expansive approach in terms of language inclusion and consideration of grey literature, to build upon and broaden the findings presented here.

Despite these potential limitations, we believe this review to also posit several strengths. First, this is the first scoping review on adult ADHD to include user representatives, which provided useful feedback on important issues expressed by members of the Norwegian ADHD user organization. Second, the developed search strategy comprised a wide variety of concepts related to psychoeducation and was applied in seven databases. Third, this is the first scoping review of psychoeducation in adult ADHD done in accordance with well-established scoping review methodology and reporting guidelines.

### Implications for future research and practice

Our findings, and the discussion above, directly lead to several implications for future research and practice. Reviews have stated that both psychoeducation and other non-pharmacological interventions show promising results [12, 13]. However, there is a need to conduct rigorous comparative trials to evaluate which elements of interventions lead to the change in outcome, as most comparative studies find positive changes in both groups with changes within groups less frequent [12, 13]. Does the information itself (or certain kinds of information) have specific effects or is it the non-specific effects of the intervention that is responsible for most of the outcome (i.e., participation effects, meeting people with similar struggles in a group, etc.)? Only one of the included interventions was digital. If learning about ADHD has specific effects, digital interventions could, in theory, have advantages in terms of scalability, standardization, reach, and availability. Such solutions may therefore also be cost-effective. It may also serve as a standardized ‘first response’ right after diagnosis before evaluating the need for more comprehensive interventions. As such, more research in digital interventions is needed.

To make such examinations possible, however, a consensus is needed on the definition of psychoeducation. Currently, as no universal definition exist, it is hard to separate psychoeducation from other psychosocial interventions. Additionally, there are no standardized guidelines regarding the information that should be given to adults with ADHD after receiving the diagnosis, and few structured manuals of more comprehensive psychoeducation interventions exist. Co-creating guidelines, interventions, and manuals with end users may aid in reaching a consensus, providing valuable feedback already at the development stage, transforming the entire research process from top-down knowledge transfer to interactive knowledge production [58]. The results from this review, however, found only one intervention, PEGASUS, that included end users both in development and delivery. After an intervention is developed, validated measures of the patient experience could guide evaluation, modification, and further implementation. Widely used, or standard measures in this area, however, are limited. The creation of future manuals should also be sensitive to traditionally unexplored areas of the diagnosis, such as sexual health and gender-specific issues and experiences.

## Conclusions

This scoping review provides an overview of the current literature on psychoeducation interventions for adults with ADHD. There is significant overlap in terms of content, but emphasis differs– with the most common themes being information about symptoms, causes, treatment options, and coping. Only one digital intervention study was included, indicating that few digital interventions are devoted to psychoeducation only. There is an urgent need for rigorous research to determine the specific and non-specific effects of these interventions, as this is still an open question. To achieve this, it is essential to develop a common understanding of what ‘psychoeducation’ means, as well as creating standardized manuals. Involving of end users in the development and delivery of interventions, and attending to the patient experience, may provide valuable feedback at all stages in these examinations. Results from this review, however, indicate that such practices are rare.

### Electronic supplementary material

Below is the link to the electronic supplementary material.

**Supplementary Material 1**: Articles excluded at the full-text stage

**Supplementary Material 2**: PRISMA-ScR Checklist

**Supplementary Material 3**: Search strategies

## Data Availability

All data generated or analysed during this review are included in this published article and its supplementary information files.

## Abbreviations

ADHD: Attention deficit hyperactivity disorder
AT: Attention training
CBT: Cognitive behavioural therapy
GMT: Goal management training
GMT+: Goal management training and psychoeducation
MAP: Mindfulness training
PE: Psychoeducation
PRISMA-ScR: The Preferred Reporting Items for Systematic reviews and Meta-Analyses extension for Scoping Reviews
RCT: Randomised controlled trial
SAT: Self alert training
SO: Significant other
TAU: Treatment as usual
