# Supplementary material for: Psychoeducation for adult ADHD: a scoping review about characteristics, patient involvement, and content
Source: BMC Psychiatry. 2024 Jan 25;24:73. doi: 10.1186/s12888-024-05530-8 (PMC10811906; doi:10.1186/s12888-024-05530-8)
Supplement: Supplementary file 1 — Supplementary Material 1: Articles excluded at the full-text stage [file 12888_2024_5530_MOESM1_ESM.docx]

**Not Psychoeducation n = 6**

Cellucci, T., Remsperger, P., & McGlade, E. (2007). Psycho-educational evaluations for university students in one clinic. *Psychological reports*, *101*(2), 501-511.

Prevatt, F., Smith, S. M., Diers, S., Marshall, D., Coleman, J., Valler, E., & Miller, N. (2017). ADHD coaching with college students: Exploring the processes involved in motivation and goal completion. *Journal of College Student Psychotherapy*, *31*(2), 93-111.

Safren SA, Sprich S, Mimiaga MJ, Surman C, Knouse L, Groves M, et al. Cognitive behavioral therapy vs relaxation with educational support for medication-treated adults with ADHD and persistent symptoms: a randomized controlled trial. Jama. 2010;304(8):875-80.

Sehlin, H., Ahlström, B. H., Bertilsson, I., Andersson, G., & Wentz, E. (2020). Internet-based support and coaching with complementary clinic visits for young people with attention- deficit/hyperactivity disorder and autism: Controlled feasibility study. *Journal of medical Internet research*, *22*(12), e19658.

Vasko JM, Meinzer MC, Murphy JG, Oddo LE, McCauley KL, Rooney ME, et al. Brief Intervention to Reduce Problem Drinking in College Students With ADHD. Cognitive and Behavioral Practice. 2019;26(3):506-21.

Wiggins D, Singh K, Getz HG, Hutchins DE. Effects of brief group intervention for adults with attention deficit/hyperactivity disorder. Journal of Mental Health Counseling. 1999;21(1):82-92.

**Conference paper n = 1**

Hirvikoski et al., (2015) Psychoeducational groups for adults with ADHD and their significant others (PEGASUS): an open clinical feasibility trial; DOI: 10.1007/s12402-015-0169-y

**Protocol study n = 1**

Pheh et al (2021): Effectiveness of online mindfulness-based intervention (Imbi) on inattention, hyperactivity–impulsivity, and executive functioning in college emerging adults with attention-deficit/hyperactivity disorder: A study protocol

**Theoretical article n = 1**

Bemporad, J., & Zambenedetti, M. (1996). Psychotherapy of adults with attention-deficit disorder. *The Journal of psychotherapy practice and research*, *5*(3), 228.
