# Supplementary material for: Psychoeducation for adult ADHD: a scoping review about characteristics, patient involvement, and content
Source: BMC Psychiatry. 2024 Jan 25;24:73. doi: 10.1186/s12888-024-05530-8 (PMC10811906; doi:10.1186/s12888-024-05530-8)
Supplement: Supplementary file 3 — Supplementary Material 3: Search strategies [file 12888_2024_5530_MOESM3_ESM.docx]

**Supplementary material 3: Search strategies**

**MEDLINE**

Ovid MEDLINE(R) ALL <1946 to May 24, 2022>

| **#** | **Searches** |
| --- | --- |
| 1 | psychoeducat*.ti,ab,kf. |
| 2 | "psycho educat*".ti,ab,kf. |
| 3 | or/1-2 [Concept #1: Psychoeducation] |
| 4 | "Attention Deficit and Disruptive Behavior Disorders"/ |
| 5 | exp Attention Deficit Disorder with Hyperactivity/ |
| 6 | exp Hyperkinesis/ |
| 7 | ((attention* or inattent* or impuls* or defian* or opposition* or disruptive* or hyperactive* or "hyper active*" or hyperkine* or "hyper kine*" or "minimal brain" or conduct) adj3 (disorder? or syndrome* or behavi?r? or deficit* or deficien* or function*)).ti,ab,kf. |
| 8 | ADHD.ti,ab,kf. |
| 9 | ADDH.ti,ab,kf. |
| 10 | ADHS.ti,ab,kf. |
| 11 | "AD HD".ti,ab,kf. |
| 12 | HKD.ti,ab,kf. |
| 13 | ODD.ti,ab,kf. |
| 14 | TOC.ti,ab,kf. |
| 15 | TDAH.ti,ab,kf. |
| 16 | or/4-15 [Concept #2: Attention Deficit Disorders] |
| 17 | and/3,16 [Concept #1 and #2 combined] |

**Cochrane Library**

ID Search Hits

#1 MeSH descriptor: [Patient Education as Topic] explode all trees

#2 (patient* NEAR/3 educat*):ti,ab,kw (Word variations have been searched)

#3 psychoeducat*:ti,ab,kw

#4 psycho-educat*:ti,ab,kw

#5 #1 OR #2 OR #3 OR #4

#6 MeSH descriptor: [Attention Deficit and Disruptive Behavior Disorders] this term only

#7 MeSH descriptor: [Attention Deficit Disorder with Hyperactivity] explode all trees

#8 ((attention* or inattent* or impuls* or defian* or opposition* or disruptive* or hyperactive* or hyper-active* or hyperkine* or hyper-kine* or minimal-brain or conduct) NEAR/3 (disorder? or syndrome* or behavio?r? or deficit* or deficien* or function*)):ti,ab,kw

#9 ADHD:ti,ab,kw

#10 ADDH:ti,ab,kw

#11 ADHS:ti,ab,kw

#12 AD-HD:ti,ab,kw

#13 HKD:ti,ab,kw

#14 ODD:ti,ab,kw

#15 TOC:ti,ab,kw

#16 TDAH:ti,ab,kw

#17 #6 OR #7 OR #8 OR #9 OR #10 OR #11 OR #12 OR #13 OR #14 OR #15 OR #16

#18 #5 AND #17

**Embase**

Embase <1974 to 2022 June 07>

| **#** | **Searches** |  |  |  |
| --- | --- | --- | --- | --- |
| 1 | exp patient education/ |  |  |  |
| 2 | (patient? adj3 educat*).ti,ab,kw. |  |  |  |
| 3 | psychoeducat*.ti,ab,kw. |  |  |  |
| 4 | "psycho educat*".ti,ab,kw. |  |  |  |
| 5 | or/1-4 [Concept #1: Psychoeducation] |  |  |  |
| 6 | exp attention deficit disorder/ |  |  |  |
| 7 | ((attention* or inattent* or impuls* or defian* or opposition* or disruptive* or hyperactive* or "hyper active*" or hyperkine* or "hyper kine*" or "minimal brain" or conduct) adj3 (disorder? or syndrome* or behavio?r? or deficit* or deficien* or function*)).ti,ab,kw. |  |  |  |
| 8 | ADHD.ti,ab,kw. |  |  |  |
| 9 | ADDH.ti,ab,kw. |  |  |  |
| 10 | ADHS.ti,ab,kw. |  |  |  |
| 11 | "AD HD".ti,ab,kw. |  |  |  |
| 12 | HKD.ti,ab,kw. |  |  |  |
| 13 | ODD.ti,ab,kw. |  |  |  |
| 14 | TOC.ti,ab,kw. |  |  |  |
| 15 | TDAH.ti,ab,kw. |  |  |  |
| 16 | or/6-15 [Concept #2: Attention Deficit Disorders] |  |  |  |
| 17 | and/5,16 [Concept #1 and #2 combined] |  |  |  |

**PsycINFO**

APA PsycInfo <1987 to May Week 5 2022>

| **#** | **Searches** |  |  |  |
| --- | --- | --- | --- | --- |
| 1 | exp Client Education/ |  |  |  |
| 2 | (patient? adj3 educat*).ti,ab,id. |  |  |  |
| 3 | psychoeducat*.ti,ab,id. |  |  |  |
| 4 | "psycho educat*".ti,ab,id. |  |  |  |
| 5 | or/1-4 [Concept #1: Psychoeducation] |  |  |  |
| 6 | exp Attention Deficit Disorder/ |  |  |  |
| 7 | exp Attention Deficit Disorder with Hyperactivity/ |  |  |  |
| 8 | exp Hyperkinesis/ |  |  |  |
| 9 | ((attention* or inattent* or impuls* or defian* or opposition* or disruptive* or hyperactive* or "hyper active*" or hyperkine* or "hyper kine*" or "minimal brain" or conduct) adj3 (disorder? or syndrome* or behavio?r? or deficit* or deficien* or function*)).ti,ab,id. |  |  |  |
| 10 | ADHD.ti,ab,id. |  |  |  |
| 11 | ADDH.ti,ab,id. |  |  |  |
| 12 | ADHS.ti,ab,id. |  |  |  |
| 13 | "AD HD".ti,ab,id. |  |  |  |
| 14 | HKD.ti,ab,id. |  |  |  |
| 15 | ODD.ti,ab,id. |  |  |  |
| 16 | TOC.ti,ab,id. |  |  |  |
| 17 | TDAH.ti,ab,id. |  |  |  |
| 18 | or/6-17 [Concept #2: Attention Deficit Disorders] |  |  |  |
| 19 | and/5,18 [Concept #1 and #2 combined] |  |  |  |

**Web of Science**

| 1 | "patient?" NEAR/3 "educat*" (Topic) |
| --- | --- |
| 2 | "psychoeducat*" (Topic) |
| 3 | "psycho educat*" (Topic) |
| 4 | #3 OR #2 OR #1 |
| 5 | ("attention*" or "inattent*" or "impuls*" or "defian*" or "opposition*" or "disruptive*" or "hyperactive*" or "hyper active*" or "hyperkine*" or "hyper kine*" or "minimal brain" or "conduct") NEAR/2 ("disorder?" or "syndrome*" or "behavio?r?" or "deficit*" or "deficien*" or "function*") (Topic) |
| 6 | "ADHD" (Topic) |
| 7 | "ADDH" (Topic) |
| 8 | "ADHS" (Topic) |
| 9 | "AD HD" (Topic) |
| 10 | "HKD" (Topic) |
| 11 | "ODD" (Topic) |
| 12 | "TOC" (Topic) |
| 13 | "TDAH" (Topic) |
| 14 | #5 OR #6 OR #7 OR #8 OR #9 OR #10 OR #11 OR #12 OR #13 |
| 15 | #4 AND #14 |

**AMED**

AMED (Allied and Complementary Medicine) <1985 to May 2022>

| **#** | **Searches** |  |  |  |
| --- | --- | --- | --- | --- |
| 1 | exp Patient education/ |  |  |  |
| 2 | (patient? adj3 educat*).ti,ab,et. |  |  |  |
| 3 | psychoeducat*.ti,ab,et. |  |  |  |
| 4 | "psycho educat*".ti,ab,et. |  |  |  |
| 5 | or/1-4 [Concept #1: Psychoeducation] |  |  |  |
| 6 | exp Attention deficit disorder with hyperactivity/ |  |  |  |
| 7 | exp Hyperkinesis/ |  |  |  |
| 8 | ((attention* or inattent* or impuls* or defian* or opposition* or disruptive* or hyperactive* or "hyper active*" or hyperkine* or "hyper kine*" or "minimal brain" or conduct) adj3 (disorder? or syndrome* or behavio?r? or deficit* or deficien* or function*)).ti,ab,et. |  |  |  |
| 9 | ADHD.ti,ab,et. |  |  |  |
| 10 | ADDH.ti,ab,et. |  |  |  |
| 11 | ADHS.ti,ab,et. |  |  |  |
| 12 | "AD HD".ti,ab,et. |  |  |  |
| 13 | HKD.ti,ab,et. |  |  |  |
| 14 | ODD.ti,ab,et. |  |  |  |
| 15 | TOC.ti,ab,et. |  |  |  |
| 16 | TDAH.ti,ab,et. |  |  |  |
| 17 | or/6-16 [Concept #2: Attention Deficit Disorders] |  |  |  |
| 18 | and/5,17 [Concept #1 and #2 combined] |  |  |  |

**ClinicalTrials.gov**

("attention*" OR "inattent*" OR "impuls*" OR "defian*" OR "opposition*" OR "disruptive*" OR "hyperactive*" OR "hyper active*" OR "hyperkine*" OR "hyper kine*" OR "minimal brain") AND ("patient education" OR "psychoeducat*" OR "psyco educat*")
